# Supplementary material for: Sequence-based model of gap gene regulatory network
Source: BMC Genomics. 2014 Dec 19;15(Suppl 12):S6. doi: 10.1186/1471-2164-15-S12-S6 (PMC4303948; doi:10.1186/1471-2164-15-S12-S6)
Supplement: Additional file 1 — Supporting information. Positional weight matrices used to predict TFBS, positions of predicted binding sites in the regulatory regions of gap genes and lists of the binding sites with regulatory weight. [file 1471-2164-15-S12-S6-S1.PDF]

Sequence-based model of gap gene regulatory network. Supporting information

Konstantin Kozlov, Vitaly Gursky,
Ivan Kulakovskiy and Maria Samsonova

Positional weight matrices

The following PWMs from [1] were used to predict TFBS.

Table 1: PWM for Hb

#	A	C	G	T
1	0.28286611155278	0.592185569462781	-0.136145867625823	-1.76751293532056
2	0.254213296764319	-0.279058161264544	0.435320237208231	-0.718027195298608
3	-3.04079579883957	0.805679778784623	-0.405565047598881	0.250334015567387
4	1.15434963541116	-2.77466891653729	-1.26870528382949	-2.98624111976211
5	1.18718322170129	-2.77466891653729	-2.77466891653729	-2.24344024824139
6	1.18360077649416	-2.65801879597414	-2.77466891653729	-2.1881892028528
7	1.17359485702533	-2.37743934902031	-2.10122450007097	-2.48109811213667
8	1.18891714677379	-2.55935994884054	-2.38229575732011	-2.69963660618354
9	1.09122776392601	-2.16244335534928	-1.62110033494844	-1.31949993562226
10	0.479767371040848	0.413371571223803	-0.553494913191296	-1.13877350496109

Table 2: PWM for Kr

#	A	C	G	T
1	-0.420658339631937	-0.371888097583974	-0.702486856000219	0.665266932827343
2	0.816288527127745	-0.672679826920774	-1.14801634271482	-0.505621250189625
3	1.02514426430819	-0.659463622858145	-2.13702100022947	-1.51520417186872
4	-2.98179845678291	1.32431226437922	-1.87626922286546	-0.620462140165821
5	-1.93735521182007	1.32865041221736	-3.92286969303831	-0.633594128997328
6	-1.76115062471767	1.48853589687916	-3.92286969303831	-3.92286969303831
7	-1.14509405073366	-0.512401154549437	-0.254529726072137	0.762459228355517
8	-1.86579042865168	-2.91908115523225	-1.65663759835415	1.144407069012
9	-3.92286969303831	-0.712178948156372	-3.92286969303831	1.12478690657717
10	-0.936923579599363	0.356401389609112	-0.354030327834424	0.41356829006083
11	0.00312114823627334	-0.123510197304167	0.564176880019257	-0.647751744510587

Table 3: PWM for Gt

#	A	C	G	T
1	0.894743440935813	-2.17380193256571	0.168460518942292	-2.63200259354209
2	0.0132767457861194	-2.15492400269797	-2.89647185910782	0.848580379348455
3	-0.38122444601539	-1.34683914600897	-0.252255198865707	0.706844550786703
4	1.01676970637832	-0.887225390259274	-0.821034544644687	-2.48732253283275
5	-0.714453934227034	1.04746188964819	-1.5648417062544	-0.31660790439989
6	-0.387096908885019	-1.03440516375464	1.21735219487855	-3.18110523181367
7	-1.29646067176372	0.580635090453266	-3.11497935075935	0.615626054743644
8	1.19590645027595	-2.38522993683808	-3.39498210194456	-2.56741818495132
9	1.1657010859841	-1.37216262127687	-2.75061891037223	-3.39498210194456
10	-0.0155498325382931	0.179390117389599	-0.811229269988477	0.247264151388273
11	1.08661227219172	-0.789512730987069	-2.17380193256571	-2.37805752062857
12	0.699718443614972	-0.208588895606494	-0.95528868329782	-0.544997743670269

Table 4: PWM for Kni

#	A	C	G	T
1	0.455726272247978	-0.388552410514004	0.253610849863229	-0.804635777714791
2	0.873682655596035	-1.46330989460881	-0.705451209523316	-0.607039387855736
3	1.17584282015165	-2.22548093796089	-2.28681238211023	-2.52042855490509
4	0.891508245810165	-2.28681238211023	-3.05088857212993	-0.0754907283730982
5	-0.167569112817295	0.433686088340233	-0.264711983511732	-0.0777049616073836
6	-1.12274746895884	-0.77721383498909	-0.181169180578325	0.786479804511023
7	0.794176684153964	-0.922134960951614	0.229047088467897	-3.17158656330019
8	-1.10178205017505	-1.51650811209733	1.38601387202952	-3.48703246125912
9	0.87958610611167	-2.26615431963949	-0.106882407802548	-1.11974612056739
10	-0.360256072608433	-0.54593643631425	0.804746228533211	-0.355643582125967
11	-2.01706986061156	1.43691160020516	-3.37951732164093	-1.5485124623163
12	0.977989859612983	-1.94764360025632	-0.31571613730388	-1.75191459249994
13	0.383982444624124	0.229251479074494	0.149205561377619	-1.50067863561956

Table 5: PWM for Bcd

#	A	C	G	T
1	-0.144456701841385	-1.87077494581996	1.16855734481103	-2.0999731022696
2	-1.30308247972915	-2.25617343877374	1.4136996497966	-2.37517799528534
3	0.917348244882398	0.128638861197661	-2.96357879753955	-2.34412541857431
4	-3.24360785523408	-3.06335363840947	-3.06335363840947	1.21431578153425
5	-2.42596650699202	-3.29887879330889	-3.29887879330889	1.20390064386744
6	1.02820586426514	-2.7190940037466	-1.35260324499878	-0.819932237368865
7	0.1127287152382	-0.902242812585579	0.832931680076778	-1.02203363892937

Table 6: PWM for Cad

#	A	C	G	T
1	-0.244447708833941	-0.0253389851612049	-0.864233992150475	0.508232127797719
2	-1.1762764127624	-1.98444131907814	-1.98444131907814	1.08676754198944
3	-1.08649544118683	-2.58276435352544	-1.64740685796158	1.07865545160057
4	-0.625527203052033	-1.1726928045224	-2.19883783759922	0.967252483469126
5	1.00983507534535	-2.58276435352544	-1.06450225118026	-0.866221178546061
6	-2.74915728201561	-2.58276435352544	-2.58276435352544	1.19406524514381
7	-0.750188864460693	-2.58276435352544	0.885452660984986	0.147546628120431
8	0.617383012458085	-2.6160756958472	0.720585804247287	-2.97727571988906
9	-1.79241413309929	0.622024634223462	0.15081288486018	0.0738539859475068
10	-1.09780598991776	0.713120163981726	-0.741759972965253	0.250863085428389

Table 7: PWM for Tll

#	A	C	G	T
1	0.689610567617782	-0.0452369723117365	-0.70275530984186	-0.886183012468088
2	0.883953461557162	-2.45775857113293	-0.189156750954037	-0.963542181806121
3	1.07938990849982	-0.990361870878966	-1.1667691782418	-3.53391190161659
4	1.0889874099488	-1.09696535299942	-1.16965279225695	-3.53391190161659
5	-1.14300585040928	-2.65377824075658	1.38978467928723	-1.94567641167443
6	-2.0025823039521	-0.150899998985087	-1.92008710225446	0.954814658711214
7	-3.53391190161659	1.38265999439891	-3.53391190161659	-0.725689699525035
8	1.15217258605849	-2.65377824075658	-1.75708800506002	-2.02766810971323
9	1.02179005292275	-3.53391190161659	-0.395421256300573	-1.71859348794298
10	0.546055171807387	0.09224289964789	-0.56441955157107	-0.652847519687621

Table 8: PWM for Hkb

#	A	C	G	T
1	0.212139821249037	-1.45196970854075	0.905491459609423	-1.41993550951193
2	-1.02889935148626	-1.9416251563326	0.658192013508353	0.462111083409141
3	-0.613018052196921	-2.49955348244106	1.3419207356921	-2.99930437854001
4	-2.7933874265196	-2.78806661379028	1.49560608167577	-2.57173903643642
5	-2.79817062558655	-2.50438480827794	1.49817518690456	-2.93131475313798
6	-2.04583529116605	1.45385405050599	-2.19808967776959	-2.23971753352978
7	-2.51597608407632	-2.77528549247748	1.49507371760739	-2.84730474556
8	-3.57861188747833	-3.05142906146399	-0.548080091989963	1.09412471793542
9	-3.05972180654926	-2.33137513542147	0.886752470121085	0.448587514168835
10	0.626498732671432	-1.54956778964025	0.38377563503559	-1.00787259801462

Positions of predicted binding sites in gap gene regulatory regions

The panels in Figures 1–4 show predicted binding sites for eight TFs in regulatory regions of *hb*, *Kr*, *gt* and *kni*. The light-gray boxes denote the DNase accessibility regions, and the dark-gray bars mark positions of the RedFly CREs that drive gene expression in the blastoderm. The transcribed region of the locus is marked in red. Only the sites overlapping with the DNase accessibility regions were included in the model.

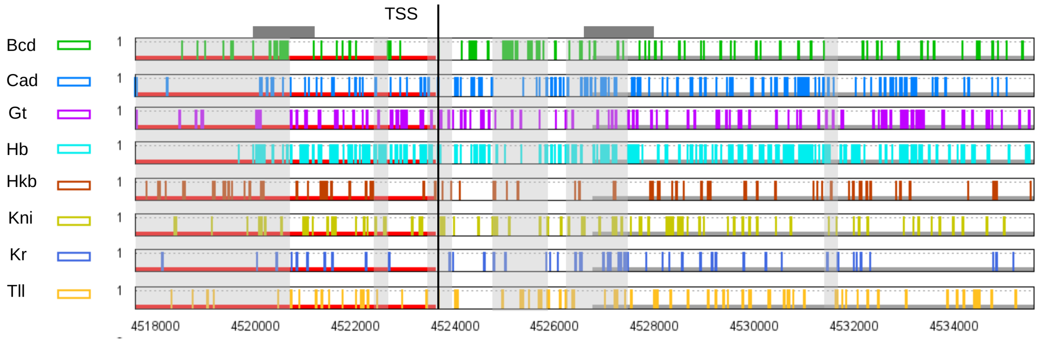

Figure 1: Predicted binding sites for TFs in *hb* regulatory region

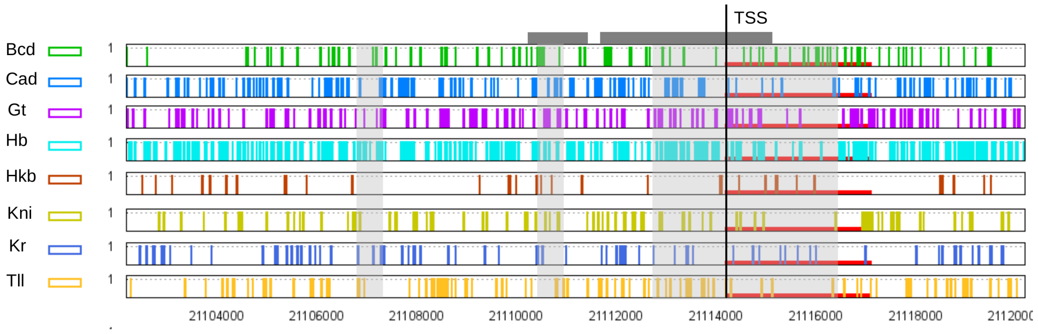

Figure 2: Predicted binding sites for TFs in *Kr* regulatory region

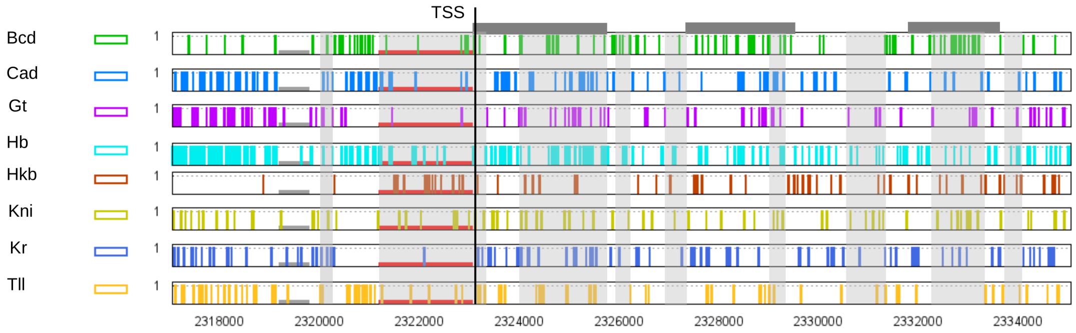

Figure 3: Predicted binding sites for TFs in *gt* regulatory region

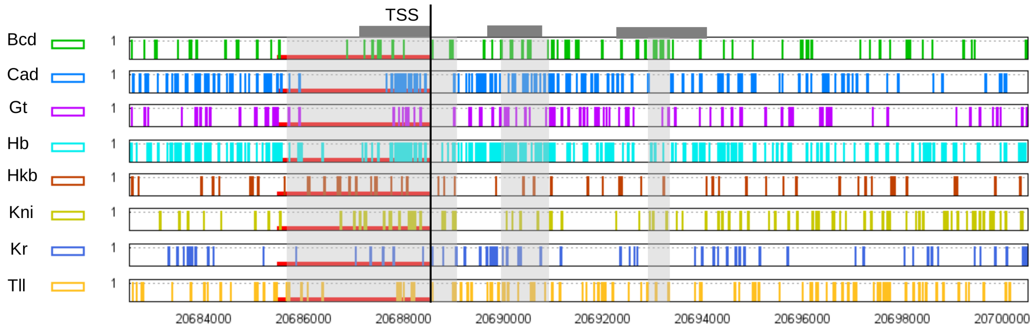

Figure 4: Predicted binding sites for TFs in *kni* regulatory region

List of binding sites with high regulatory impact in gap gene regulatory regions

Table 9: List of binding sites with high RW in *hb* regulatory region

Coordinates	TF	word	w_r	construct
4531368..4531378	hb	GTTTTTTTCCG	0.007	no
4526994..4527004	hb	GTTTTTTTGTG	0.083	hb_HZ1.4;hb_HZ526;hb_upstream_enhancer
4526954..4526964	hb	GTTTTTTATG	0.060	hb_HZ1.4;hb_HZ526;hb_upstream_enhancer
4526893..4526903	hb	CGCAAAAAAC	0.046	hb_HZ1.4;hb_HZ526;hb_upstream_enhancer
4526881..4526891	hb	CCTAAAAAAC	0.026	hb_HZ1.4;hb_HZ526;hb_upstream_enhancer
4526212..4526222	hb	AGCAAAAAAA	0.017	no
4525759..4525769	hb	GTTTTTTCTT	0.007	no
4525639..4525649	hb	TTTTTTTGTG	0.020	no
4522356..4522366	hb	GGCAAAAAAC	0.007	no
4520486..4520493	bcd	GGATTAG	0.021	hb_0.7;hb_ant_act;hb_HB161;hb_HB263;hb_HB747
4520381..4520388	bcd	GGATTAG	0.021	hb_0.7;hb_ant_act;hb_HB161;hb_HB263;hb_HB747

Table 10: List of binding sites with high RW in *Kr* regulatory region

Coordinates	TF	word	w_r	construct
21110739..21110749	tll	AGAAGTCAAA	0.008	Kr_CD1;Kr_StBg1.2HZ;Kr_StH0.6HZ;Kr_730
21110829..21110841	gt	TTCTTGCGTCAT	0.038	Kr_CD1;Kr_StBg1.2HZ;Kr_StH0.6HZ;Kr_730
21112818..21112830	gt	ATTTTACGTAAC	0.010	Kr_H/J;Kr_NcS1.7HZ
21113416..21113429	kni	CTGTTTCATTTTC	0.021	Kr_SN1.7KrZ
21113432..21113445	kni	CTGTTCTCCATTG	0.040	Kr_SN1.7KrZ
21113690..21113702	gt	TTTTTATGTTTT	0.023	Kr_H/I;Kr_SN1.7KrZ
21113698..21113708	cad	TTTTATTACT	0.047	Kr_H/I;Kr_SN1.7KrZ
21113723..21113733	cad	GGTCATAAAA	0.342	Kr_H/I;Kr_SN1.7KrZ
21113852..21113865	kni	TCGCACCAATTTG	0.021	Kr_H/I;Kr_SN1.7KrZ
21113941..21113953	gt	TTTTTACTTTAC	0.036	Kr_H/I;Kr_K/F;Kr_SN1.7KrZ

Table 11: List of binding sites with high RW in *gt* regulatory region

Coordinates	TF	word	w_r	construct
2333959..2333969	hb	GTTTTTTGTC	0.007	no
2333157..2333164	bcd	GTAATCC	0.008	gt_10;gt_gt23
2333132..2333145	kni	AAAACTGGGCCAT	0.007	gt_10;gt_gt23
2333031..2333043	gt	AATCCGTAAGAT	0.0078	gt_10;gt_gt23
2332983..2332996	kni	ATGGTCGAGTTCC	0.009	gt_10;gt_gt23
2332949..2332956	bcd	AGATTAG	0.006	gt_10;gt_gt23
2332325..2332338	kni	CTGCTCTGAATTG	0.057	gt_10;gt_gt23
2332260..2332267	bcd	GGATTAG	0.036	gt_10
2331158..2331171	kni	GTGGTCTTTTTTC	0.015	no
2329230..2329243	kni	GAAACTGGTTCTC	0.012	gt_6
2329212..2329222	hb	ATTTTGTGCG	0.009	gt_6
2329042..2329055	kni	TTGCGCCATTTTG	0.157	gt_6
2329039..2329051	gt	GGATTGCGCCAT	0.028	gt_6
2329014..2329026	gt	GTTACGTTACAT	0.009	gt_6
2329012..2329024	gt	ATGTTACGTTAC	0.467	gt_6
2327063..2327073	hb	AGCAAAAGAA	0.007	no
2325885..2325892	bcd	CTAAGCC	0.046	no
2325231..2325244	kni	CCGCTGCAGTTTT	0.439	gt_3;gt_CE8001
2325151..2325161	hb	CCTAAAAAAC	0.016	gt_3;gt_CE8001
2325099..2325110	Kr	CGAACGGGTTG	0.231	gt_3;gt_CE8001
2325070..2325080	hb	GTTTTTTACG	0.016	gt_3;gt_CE8001
2325042..2325053	Kr	CAATCCTTTGA	0.009	gt_3;gt_CE8001
2324948..2324961	kni	TAAAAAACAGCAG	0.071	gt_3;gt_CE8001
2324946..2324956	hb	CGTAAAAAAC	0.010	gt_3;gt_CE8001
2324852..2324865	kni	CGATCCCGAGCAG	0.039	gt_3;gt_CE8001
2324675..2324685	hb	AGTAAAAAAC	0.005	gt_3;gt_CE8001
2324080..2324093	kni	GAAATTAGGACGA	0.006	gt_1
2320014..2320024	hb	ttttttttatt	0.010	no

Table 12: List of binding sites with high RW in *kni* regulatory region

Coordinates	TF	word	w_r	construct
20693268..20693275	bcd	AGATTAG	0.011	kni_-5;kni_anterioventral
20693222..20693235	kni	TGATCTCGAGCGA	0.008	kni_-5;kni_anterioventral
20693164..20693174	hb	GGGAAAAAAC	0.362	kni_-5;kni_anterioventral
20693137..20693144	bcd	CTAAGCC	0.017	kni_-5;kni_anterioventral
20693107..20693114	bcd	GTAATCC	0.008	kni_-5;kni_anterioventral
20693018..20693025	bcd	GGCTTTG	0.009	kni_-5;kni_anterioventral
20692973..20692980	bcd	TAAATCC	0.008	kni_-5;kni_anterioventral
20692962..20692975	kni	CTGATCTCAATTA	0.232	kni_-5;kni_anterioventral
20692924..20692934	hb	GTTTTTCACA	0.013	kni_-5;kni_anterioventral
20692891..20692904	kni	GGAAATGCGGCAA	0.018	kni_-5;kni_anterioventral
20690707..20690718	Kr	GAAAAGGGATA	0.014	no
20690608..20690618	cad	AACCATAAAA	0.018	kni_223
20690581..20690591	cad	AGTCATAAAG	0.015	kni_223
20688973..20688985	gt	TTTTTTTGGCAT	0.018	no
20688786..20688799	kni	TCTCTCTCGATTT	0.007	no
20688736..20688749	kni	GAATAGAAAACAC	0.009	no
20688541..20688548	bcd	GGCTTAT	0.106	no
20688538..20688548	tll	TTTGGCTTAT	0.046	no
20688532..20688543	Kr	GAACTCTTTGG	0.006	kni_1.8lacZ

References

[1] I.V. Kulakovskiy and V.J. Makeev. Discovery of dna motifs recognized by transcription factors through integration of different experimental sources. *Biophysics*, 54(6):667–674, 2009.
